# Long noncoding RNA LINC00261 suppresses prostate cancer tumorigenesis through upregulation of GATA6-mediated DKK3

**DOI:** 10.1186/s12935-020-01484-5

**Published:** 2020-09-30

**Authors:** Yang Li, Hai Li, Xin Wei

**Affiliations:** Department of Urology, China-Japan Union Hospital of Jilin University, No. 126, Xiantai Street, Changchun, 130033 Jilin People’s Republic of China

**Keywords:** Prostate cancer, Long noncoding RNA LINC00261, GATA binding protein 6, Dickkopf-related protein 3

## Abstract

**Background:**

Prostate cancer is one of the leading causes of cancer death in males. Recent studies have reported aberrant expression of lncRNAs in prostate cancer. This study explores the role of LINC00261 in prostate cancer progression.

**Methods:**

The differentially expressed genes, transcription factors, and lncRNAs related to prostate cancer were predicted by bioinformatics analysis. Prostate cancer tissue samples and cell lines were collected for the determination of the expression of LINC00261 by reverse transcription quantitative polymerase chain reaction. The binding capacity of LINC00261 to the transcription factor GATA6 was detected by RIP, and GATA6 binding to the DKK3 promoter region was assessed by ChIP. In addition, luciferase reporter system was used to verify whether LINC00261 was present at the DKK3 promoter. After gain- and loss-of function approaches, the effect of LINC00261 on prostate cancer in vitro and in vivo was assessed by the determination of cell proliferation, invasion and migration as well as angiogenesis.

**Results:**

LINC00261, GATA6, and DKK3 were poorly expressed in prostate cancer. LINC00261 could inhibit transcriptional expression of DKK3 by recruiting GATA6. Overexpression of LINC00261 inhibited prostate cancer cells proliferation, migration, and invasion as well as angiogenesis, which could be reversed by silencing DKK3. Furthermore, LINC00261 could also suppress the tumorigenicity of cancer cells in vivo.

**Conclusions:**

Our study demonstrates the inhibitory role of LINC00261 in prostate cancer progression, providing a novel biomarker for early detection of prostate cancer.

## Background

Prostate cancer is a life-threatening malignancy for men with approximately 1.3 million new cases and 359,000 deaths occurred in 2018 [1]. Of note, prostate cancer is more common in developed countries than developing countries, the former also having a higher mortality rate [2]. In the past decade, the early detection of prostate cancer was improved by prostate-specific antigen (PSA), which is reflected by increased detection rate of localized tumors [3]. Aberrant expression of long noncoding RNAs (lncRNAs) plays an essential role in cancer progressions [4].

As a class of transcripts, lncRNAs are involved in various biological processes [5]. LncRNAs are often dysregulated when faced with disturbance of cellular function. Therefore, lncRNAs can be used as early markers for disease detection. A recent report showed that lncRNA HOTAIR expression was increased in castration-resistant prostate cancer cells, and HOTAIR is involved in prostate cancer cell biological functions [6]. However, the mechanism by which LINC00261 exerts its functions in prostate cancer is still unclear.

As a divergent member of the Dickkopf (DKK) family, the Dickkopf-related protein 3 (DKK3) is a tumor suppressor involved in slowing down the progressions of many types of cancer. For example, DKK3 is expressed at a low level in endometrial cancer, indicating that DKK3 can also be used as a new biomarker in endometrial cancer [7]. In addition, a recent study has shown that induction of DKK3 can inhibit prostate cancer development [8]. DKK3 could induce cellular quiescence in prostate cancer cells through activating the p38MAPK signaling pathway [9]. Further, it was noted that DKK3 mediated Wnt/β-catenin pathway and thereby suppressed in cellular processes in prostate cancer and hepatocellular carcinoma, incorporating with miRNA [10, 11]. However, the role of DKK3 that regulating by LINC00261 in the development of prostate cancer was unknown.

Furthermore, GATA binding protein has been considered as a potential therapeutic target in prostate cancer for its independent prognostic value. For example, GATA3 expression is decreased in prostate cancer cells, which promotes cell growth, colony formation, cell migration, and invasion [12]. As a single factor, GATA binding protein 6 (GATA6) is downregulated in many tumors, particularly in gastrointestinal cancer. For example, GATA6 in conjunction with KLF5 and GATA4, has been implicated in gastric cancer tumorigenesis [13]. Therefore, it is reasonable to predict GATA6 could be related to prostate cancer development. Here we explore the functional relationships between LINC00261, DKK3, and GATA6 as well as their roles in prostate cancer in order to uncover a promising strategy for prostate cancer treatment.

## Methods

### Bioinformatics analysis

The publicly available datasets for LINC00261 and DKK3 were used to predict their involvement in prostate cancer. Gene Expression Omnibus (GEO) database (https://www.ncbi.nlm.nih.gov/) was used to download the prostate cancer-related gene expression dataset GSE45016, including 10 samples of prostate cancer tissues and 1 sample of adjacent normal tissues. The limma package was used for screening the differentially expressed genes (DEGs) of prostate cancer, with |log2 fold change (FC)| > 2.0, and adj. *p* value < 0.05 as the screening criteria. By comparing the FC of LncRNA in DEGs, the lncRNA with the largest FC was selected for further study. The LncMap (http://bio-bigdata.hrbmu.edu.cn/LncMAP/index.jsp) database was used to predict the significantly differentially expressed lncRNAs and the relationship among lncRNAs, transcription factors (TF) and genes. GEPIA database (http://gepia.cancer-pku.cn/index.html) was employed to verify the expression of DEGs, including 492 cancer tissues sample and 152 normal samples. Prostate cancer-related data were obtained from The Cancer Genome Atlas (TCGA) database and the relationship between the expression of LINC00261 and DKK3 as well as the relationship between the expression of LINC00261, DKK3 and patient prognosis were analyzed.

### Sample collection

A total of 83 patients with prostate cancer were selected for sample collection. Patients meeting the following criteria were enrolled: patients confirmed by tumor pathology and genetics, patients without other histories of urologic neoplasms or tumor, and patients who received no chemotherapy or radiotherapy before the operation. Patients meeting the following criteria were excluded: patients with severe impairment of vital organs such as heart, liver, and lung; patients with autoimmune history, patients with autoimmune history, and patients with chronic infectious disease or acute contagious diseases. Another 60 cases of adjacent normal tissues were taken as negative controls (NC). There was no significant difference in age among the groups (*p* > 0.05).

### Cell treatment

Human prostate cancer cell lines LNCap, PC-3, DU145, 22Rv1, ARCaP, and normal prostate cell line RWPE-1 purchased from American Type Culture Collection (Manassas, VA, USA) were selected and cultured in Roswell Park Memorial Institute 1640 medium with 10% serum at 5% CO_2_ and 37 ℃. RT-qPCR was used to measure LINC00261 expression, and the cell line with the lowest expression was selected for the experiment. When confluence reached 80–90%, cells were subcultured. Then the cells were added with 200 µl 0.25% ethylenediaminetetraacetic acid trypsin, placed in the incubator for 2–5 min, and observed under the inverted microscope. When cell shrinkage and cell gap enlargement were observed, the culture was terminated immediately by adding 1 mL of culture medium with 10% serum. Subsequently, the cells were made into cell suspension and cultured at 37℃ and 5% CO_2_.

According to the NCBI, the sequences of LINC00261, GATA6, and DKK3 were obtained. Shanghai Sangon Biotechnology Co. Ltd. (Shanghai, China) was entrusted to construct the interference and overexpression sequences of LINC00261, GATA6, and DKK3. The cells were transfected with the plasmids of DKK3 overexpression (oe-DKK3), siRNA targeting DKK3 (si-DKK3), oe-LINC00261 and si-GATA6 alone or in combination according the instructions of lipofectamine 2000 (11668-019, Initrogen, New York, California, USA).

### RNA isolation and quantitation

Total RNA was extracted from tissues and transfected cells with Trizol using the microRNA Neasy Mini Kit I (217,004, Qiagen company, Hilden, Germany). The primers for LINC00261, GATA6, and DKK3 were synthesized by Takara Bio Inc. (Otsu, Shiga, Japan) (Table 1). The RNA was reverse transcribed by the PrimeScript RT kit (RR036A, Takara Bio Inc., Otsu, Shiga, Japan). Fluorescence quantitative PCR was performed with the SYBR® Premix ExTaq™ II kit (RR820A, Takara Bio Inc., Otsu, Shiga, Japan) on an ABI PRISM® 7300 system (Prism® 7300, Shanghai Kunke Co., Ltd., Shanghai, China). β-Actin was used as the internal reference for LINC00261, GATA6, and DKK3. The relative expression of each target gene was calculated by 2^−ΔΔCt^ method.

**Table 1:** The primer sequences

Name	Sequence (5′–3′)	
LINC00261	F: GTCAGAAGGAAAGGCCGTGA	
	R: TGAGCCGAGATGAACA GGTG	
GATA6	F: GTGAACTGCGGCTCCATCCA	
	R: CCTTCCCTTCCATCTTCTCTCA	
DKK3	F: GTAAGTTCCCCTCTGGCTTG	
	R: AAGCACCAGACTGTGAAGCCT	
β-actin	F: CGCACCACTGGCATTGTCAT	
	R: TTCTCCTTGATGT-CACGCAC	

*LINC00261* long non-coding RNA 00261, *GATE6* GATA binding protein 6, *DKK3* dickkopf-related protein 3, *F* forward, *R* reverse

### Western blot analysis

Total protein was extracted using the Radio Immunoprecipitation Assay (RIPA) lysis buffer with phenylmethylsulfonyl fluoride (R0010, Beijing Solarbio Science & Technology Co., Ltd., Beijing, China), then separated by electrophoresis and transferred onto a polyvinylidene fluoride (PVDF) membrane. After blocking with 5% skim milk for 1 h, the PVDF membrane was incubated with diluted GATA6 rabbit polyclonal antibody (1 : 1000, ab175349), DKK3 rabbit monoclonal antibody (1 : 1000, ab186409), matrix metalloproteinase 2 (MMP-2) rabbit monoclonal antibody (1 : 500, ab37150), and MMP-9 rabbit polyclonal antibody (1 : 1000, ab38898), vascular endothelial growth factor (VEGF) rabbit monoclonal antibody (1 : 1000, ab32152), and CD31 mouse monoclonal antibody (1 : 1000, ab9498) overnight at 4℃. All antibodies were purchased from Abcam Inc. (Cambridge, UK). Subsequently, the membrane was incubated with horseradish peroxidase (HRP) conjugated secondary antibody goat anti-mouse IgG (HA1003, Shanghai Yanhua Biotechnology Co., Ltd., Shanghai, China) for 1 h. The membrane was treated with enhanced chemiluminescence (ECL) solution (ECL 808 − 25, Biomiga, Inc., San Diego, California, USA) for 1 min and observed after taking X-ray (36209ES01, Qianchen Biotechnology Co., Ltd., Shanghai, China). β-actin was used as the internal reference.

### Fluorescence in situ hybridization (FISH)

The promoter region of DKK3 was connected into the pGL3-basic vector (Promega, Madison, WI, USA) to form the pGL3-DKK3 recombinant vector. HEK293T cells were plated into a 24-well plate at a density of 3 × 10^4^ cells/well. pGL3-DKK3 was co-transfected with oe-GATA6 and si-GATA6, respectively. After 48 h, activation of the target reporter gene was analyzed by a dual-luciferase reporter gene analysis system (Promega Corporation, Madison, WI, USA).

The subcellular localization of LINC00261 in prostate cancer cells was identified by FISH, and the results were analyzed by the Biological Prediction Website. According to the instructions of Ribo™ lncRNA FISH Probe Mix (Red) (Guangzhou RiboBio Co., Ltd., Guangdong, China), the specific methods were as follows [14]: cells were cultured into a 24-well plate at 6 × 10^4^ cells/well until they reached 80% confluence. Next, the cells were fixed with 1 mL 4% polyformaldehyde. After treatment with protease K (Beijing Solabio Life Sciences Co., Ltd, Beijing, China) (2 µg/mL), glycine and ethyl phthalide reagent, 250 µl pre-hybridization solution was added for incubation at 42℃ for 1 h. After that, 250 µl hybridization solution containing the probe (300 ng/mL) was added and hybridized overnight at 42℃. The cell nucleus was stained with 4,6-diamino-2-phenyl indole (DAPI) diluted by Phosphate-Buffered Saline/Tween in the 24-well plate for 5 min and then sealed with the anti-fluorescence quenching agent. At last, five different visual fields were selected for observation and photography under a fluorescence microscope (Olympus Optical Co., Ltd, Tokyo, Japan).

### 5-Ethynyl-2′-deoxyuridine (EdU) assay

The cell culture plates of each group were treated with EdU solution and incubated for two h. After fixation with 40 g/l polyformaldehyde for 30 min and incubation with glycine solution for 5 min, the cells were cleaned with Phosphate-Buffered Saline (PBS) containing 0.5% Triton X-100. Subsequently, the cells were treated with Apollo® staining solution and incubated for 30 min in the dark. After washing with formaldehyde and PBS, Hoechst 3334 solution was added to the cells which were then incubated for 30 min in the dark, and observed under the fluorescence microscope. Three visual fields were selected out of 400 visual fields, and the EdU stained cells (proliferating cells) and Hoechst 3334 stained cells (total cells) were counted [15].

### Transwell assay for cell migration and invasion assessment

Cells were treated with trypsin and plated into the apical chamber of a 24-well plate after the cell density was adjusted to 1 × 10^6^ cells/mL using serum-free H-Dulbecco’s modified eagle’s medium (DMEM) and L-DMEM medium, and the basolateral chamber of the 24-well plate added with H-DMEM and L-DMEM medium with 10% FBS beforehand. Cells were incubated in the apical chamber for 12 h for migration assessment. For cell invasion assessment, after transfection for 48 h, cells were coated with Matrigel (1 : 10, No. 356,234, Becton, Dickinson and Company, NJ, USA) and incubated at 37℃ for 24 h. Cells that did not migrate were removed with a cotton swab and migratory cells were fixed with 5% glutaraldehyde at 4℃ and stained with crystal violet for 30 min. Observation was conducted under the microscope. The number of cells passing through the matrix gum in each group was used as an index to evaluate their invasive ability [16].

### Chromatin immunoprecipitation (ChIP)

The cells were fixed with 16% formaldehyde and cross-linked. Then, cells were lysed by cell lysate and broken by the ultrasonic wave and added with 5 µg GATA6 (Cat_AF1700, R&D Systems, Shanghai, China) antibody for overnight incubation. After that, magnetic beads were added to capture protein-DNA binding complexes and then washed with washing buffer. A total of 5 mol/L NaCl was added into the cells for de-cross-linking, and then the DNA was collected. The binding of the DKK3 promoter in the complexes was assessed by fluorescent PCR [17].

### RNA immunoprecipitation (RIP)

The binding of LINC00261 to GATA6 was assessed using the RIP Kit (Millipore, Billerica, MA, USA). Cells were lysed with RIPA lysis buffer (P0013B, Beyotime Biotechnology Co., Shanghai, China) on ice for 5 min and centrifuged at 140 000 rpm for 10 min at 4℃. One part of the cell extracts was taken out as input, and the other part was incubated with the antibody for coprecipitation. The specific steps were as follows: 50 µl magnetic beads were resuspended in 100 µl RIP Wash Buffer in each coprecipitation reaction system. Then, 5 µg antibody was added for binding. The magnetic bead-antibody complex was resuspended in 900 µl RIP Wash Buffer and incubated overnight at 4℃ with 100 µl of cell extract. Samples were placed on a magnetic base to collect the bead-protein complexes. RNA was extracted from the samples and the input, after treatment with protease K, LINC00261 expression was measured by PCR [18]. The antibodies used were GATA6 (Cat_AF1700, R&D Systems, Shanghai, China), and IgG (ab109489, 1 : 100) as NC.

### Tube formation in vitro

The Matrigel was frozen and thawed overnight at 4℃. Next, 75 µl Matrigel was added to each well of a pre-cooled 96-well plate which was then placed at 37℃ for 60 min. The suspension of human microvascular endothelial cells and human umbilical vein endothelial cells (HUVECs) was added to the 96-well plate at a concentration of 2.5 × 10^4^ cells/well. After cells adhered to the wall, the culture medium was replaced by the supernatant of the transfected prostate cancer cells and incubation was continued for 4–6 h, followed by observation and photography under a microscope [19].

### Xenograft tumor in nude mice

Twenty-four male BALB/c-nu/nu nude mice (specific pathogen free grade, 5-week-old, Shanghai SLAC Laboratory Animal Co., Ltd, Shanghai, China) were treated with the following plasmids: oe-LINC00261, sh-DKK3, and sh-GATA6 alone or in combination. The stably transfected LNCap cell line of prostate cancer was diluted to a cell density of 2.5 × 10^7^ cells/mL. And 200 µl cells were subcutaneously inoculated into the neck and back of nude mice. Tumor formation was monitored weekly and the tumor volume (TV) and maximum (a) and minimum (b) diameters of transplanted tumor nodules in nude mice were measured. On the 30th d, the nude mice were euthanized and tumors were collected and prepared for paraffin sections.

### Immunohistochemistry

Paraffin-embedded sections were routinely deparaffinized with Xylene I and II (each for 10 min), dehydrated with gradient ethanol, immersed in 3% hydrogen peroxide for 10 min, repaired with high-pressure antigen for 90 s, sealed with 5% bovine serum albumin (BSA), and incubated for 30 min at 37 ℃. Then 50 µl DKK3 rabbit anti-mouse monoclonal antibody (1:1000, ab186409, Abcam Inc., Cambridge, UK) was added and incubated overnight at 4℃. After 2 min of washes with PBS, 50 µl biotinylated mouse anti-goat IgG (RXE0155, Shanghai Rongchuang Biotechnology Co., Ltd., Shanghai, China, 1:100) was added for incubation for 30 min at 37 ℃. The cells were then stained with diaminobenzidine (DAB) and re-stained with hematoxylin for 5 min. PBS buffer was introduced into this procedure as the negative control slides. After conventional treatment, five high power visual fields were randomly selected to observe the positive expression rates of VEGF and CD31.

### Statistical analysis

Measurement data were presented as mean ± standard deviation. The data conforming to the normal distribution and homogeneous variance between two groups were analyzed by paired (for paired data) or unpaired *t*-test (for unpaired data). Comparisons among multiple groups were analyzed using the one-way analysis of variance (ANOVA) with Tukey’s post hoc test used. The data at different time points were analyzed by the repeated measures ANOVA followed by Bonferroni’s post hoc test. A value of *p* < 0.05 was considered statistically significant.

## Results

### The significance of LINC00261 in prostate cancer

**Fig. 1:**
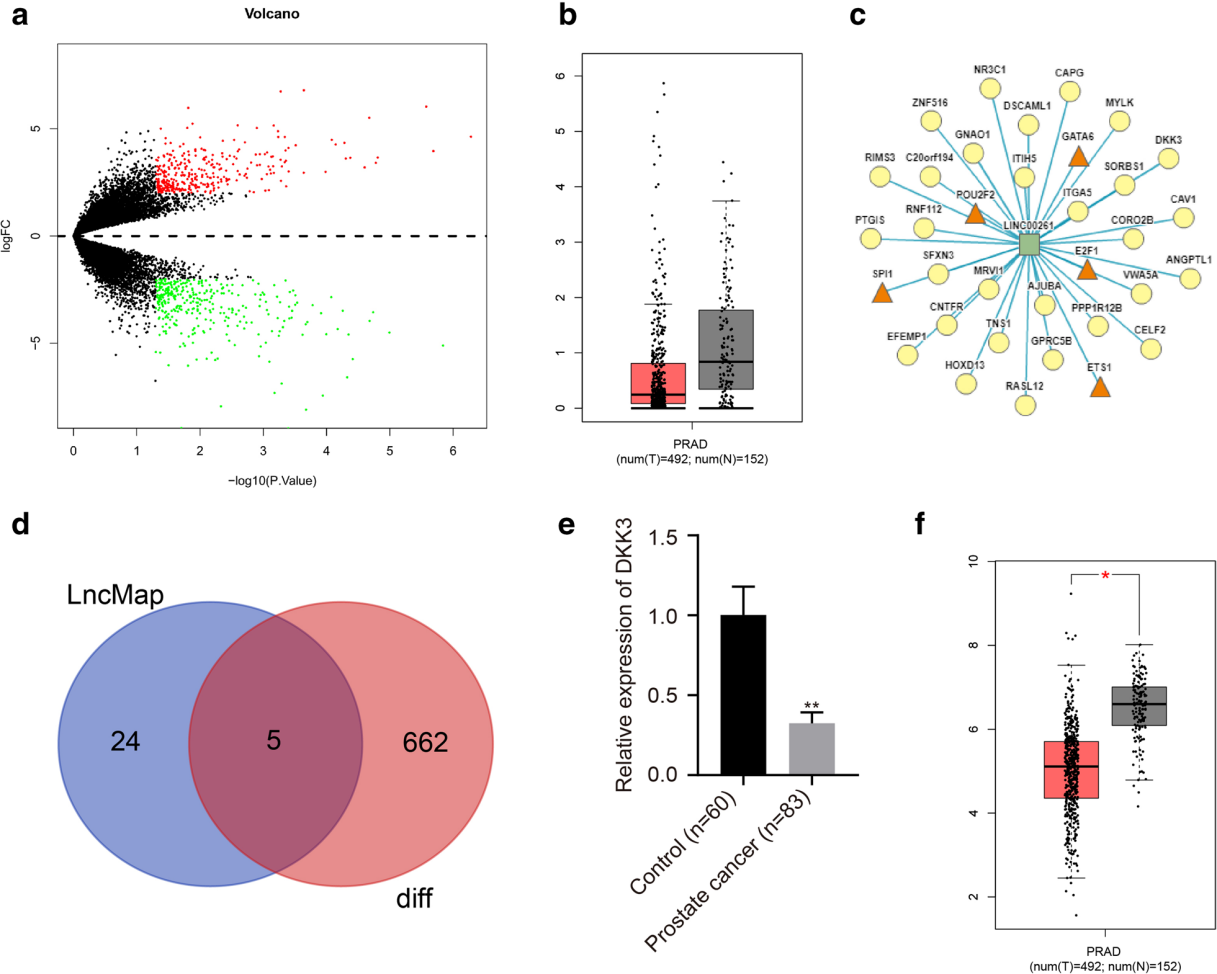
LINC00261 binds to the transcription factor GATA6 to regulate DKK3. **a** The Volcano map of expression of DEGs in prostate cancer. LogFC is represented on the x axis and log10 *p* value on the y axis. Each point in the graph represents a gene, red dot represents an up-regulated gene, green dot represents a down-regulated gene. **b** The expression of LINC00261 in prostate cancer tissues and normal samples in TCGA database, The x axis indicates the disease name and the number of samples, the y axis indicates the expression value, the red box graph represents the tumor sample, and the gray box graph represents the normal sample. **c** The relationship among lncRNA, transcription factor and gene. **d** The intersection of genes in LncMap database and DEGs in microarray GSE45016. The circle on the left represents the prediction results of the LncMap database, the circle on the right represents the differential genes in the new product, and the middle part represents the intersection of the two sets of data. **e** The expression of DKK3 in prostate cancer and normal tissues assayed by RT-qPCR, *p < 0.05 vs. the normal tissues, **p < 0.01 vs. the normal tissues. **f** The expression of DKK3 in prostate cancer and normal tissues in TCGA database. The x axis indicates the disease name and the number of samples, the y axis indicates the expression value, the red box graph represents the tumor sample, and the gray box graph represents the normal sample

The gene expression dataset GSE45016 was downloaded from the GEO database, and 667 prostate cancer-related DEGs were obtained by differential analysis, of which 329 were highly expressed and 338 poorly expressed genes (Fig. 1a). The differential expression multiples of lncRNAs were screened from the DEGs and the largest multiple LINC00261 was selected for further study (Table 2). Due to the small number of normal samples in the dataset, in order to confirm the accuracy of the analysis results, we used the Gene Expression Profiling Interactive Analysis (GEPIA) database to retrieve the expression of LINC00261 in TCGA. The GEPIA database further verified that LINC00261 is expressed at a low level in prostate cancer (Fig. 1b).

**Table 2:** Differential expression data of LncRNAs in GSE45016

	logFC	P.value
LINC00326	3.99227	0.000445
LINC00948	2.915857	0.00076
LINC00261	− 5.71112	0.005246
LINC00165	3.551828	0.00851
LINC01118	− 2.18818	0.009387
LINC01116	3.973839	0.011807
LINC01234	2.850941	0.018537
LINC00964	− 4.53925	0.019741
LINC00309	2.842735	0.024328
LINC01139	− 2.90076	0.025123
LINC01243	3.772044	0.028488
LINC01410	− 2.67942	0.03061
LINC00673	3.2997	0.036729
LINC00507	− 3.85766	0.038307
LINC01069	2.548307	0.039803
LINC00327	− 2.5148	0.043816
LINC00605	2.652351	0.045137

The LncMap database revealed that the genes could be regulated by LINC00261 through a transcription factor (Fig. 1c). According to the DEG analysis and the GSE45016 microarray data predicted by the LncMap database, five DEGs (DKK3, HOXD13, CAPG, ANGPTL1, and EFEMP1) were obtained (Fig. 1d). DKK3 was verified to be the most differentially expressed gene which was selected for further study (Fig. 1e). According to the LncMap prediction, LINC00261 regulated DKK3 through the transcription factor GATA6. Furthermore, validation with GEPIA database revealed that DKK3 expression was also decreased in prostate cancer (Fig. 1f), which further demonstrated that LINC00261 might regulate DKK3 by binding to transcription factor GATA6, thus affecting the development of prostate cancer.

### LINC00261 expression is reduced in prostate cancer tissues and cells

**Fig. 2:**
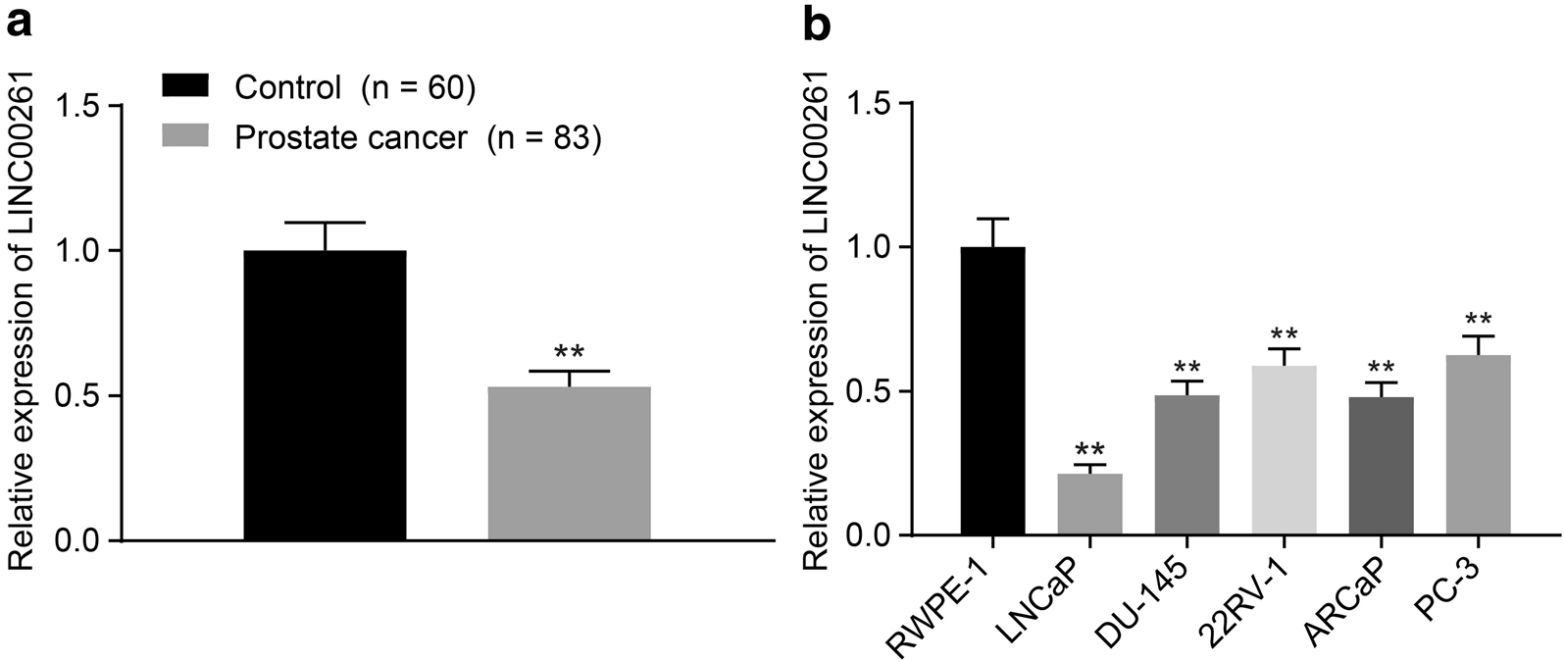
Expression of LINC00261 was decreased in prostate cancer tissues and cells. **a** Comparison of the LINC00261 expression in normal and cancer tissues, n = 83, *p < 0.05 vs. the normal tissues, **p < 0.01 vs. the normal tissues. **b** Comparison of the LINC00261 expression in four prostate cancer cell lines and normal prostate cells, *p < 0.05 vs. the prostate cancer cell line RWPE-1, **p < 0.01 vs. the prostate cancer cell line RWPE-1. Statistical data and measurement data are described as mean ± standard deviation. The unpaired *t* test is used to compare data between two groups. ANOVA is used to compare data among multiple groups, followed by Tukey’s post hoc test. The experiment was repeated three times

RT-qPCR results showed that compared with adjacent normal tissues, LINC00261 expression in human prostate cancer tissues was decreased (Fig. 2a, *p* < 0.05). Meanwhile, LINC00261 expression in human prostate cancer cell lines LNCap, PC-3, DU145, and 22Rv1, was declined compared to the normal prostate cell line RWPE-1, with the lowest expression found in LNCap (Fig. 2b). Thus, LNCaP was selected for subsequent experiments with the fact of lower expression of LINC00261 in prostate cancer.

### Overexpression of LINC00261 inhibits proliferation, migration, invasion and tube formation of prostate cancer cells

**Fig. 3:**
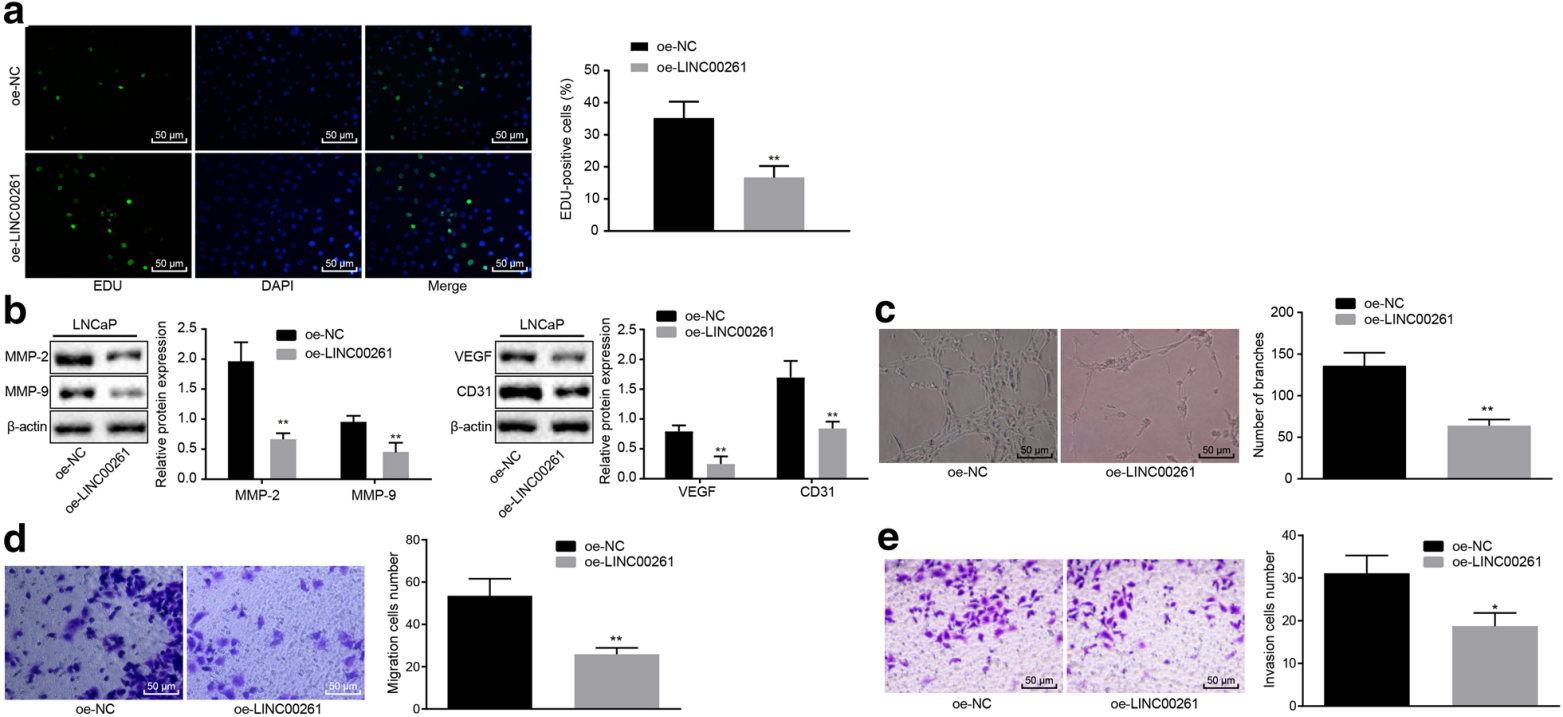
Upregulated LINC00261 suppresses the proliferation, migration, invasion, and lumen formation of prostate cancer cells. **a** Representative images and statistical analysis of proliferation detected by EdU (200 ×). **b** Grey analysis and statistical analysis of protein of MMP-2, MMP-9, VEGF, CD31, and β-actin measured by Western blot analysis **c** Representative images and statistical analysis of tube formation (×100). **d**, **e** Representative images and statistical analysis of cell migration (×200) and invasion (×200) detected by Transwell assay. * *p* < 0.05 vs. the cells treated with oe-NC, **p < 0.01 vs. the cells treated with oe-NC. Statistical data are measurement data and described as mean ± standard deviation. Unpaired *t* test is used to compare data between two groups. The experiment was repeated three times

To further investigate the effect of LINC00261 on the proliferation of prostate cancer cells, an EdU proliferation test was conducted. When compared with the treatment of oe-NC, the proliferation rate of cells transfected with oe-LINC00261 was decreased (Fig. 3a). Furthermore, Western blot analysis revealed that when compared to oe-NC-transfected cells, the protein expression of MMP-2 and MMP-9 and also VEGF and CD31 was decreased in cells transfected with oe-LINC00261 (Fig. 3b) (*p* < 0.05). The tube formation experiment showed the tube formation ability of cells treated with oe-LINC00261 was reduced compared with the cells treated with oe-NC (Fig. 3c). Furthermore, the Transwell assay demonstrated that compared with the cells treated with oe-NC, the migration and invasion ability of the cells transfected with oe-LINC00261 was decreased (Fig. 3d, e). Therefore, LINC00261 overexpression exerted an inhibitory effect on the proliferation, migration, invasion of tumor cells and tube formation ability of vascular endothelial cells.

### LINC00261 regulates DKK3 expression

**Fig. 4:**
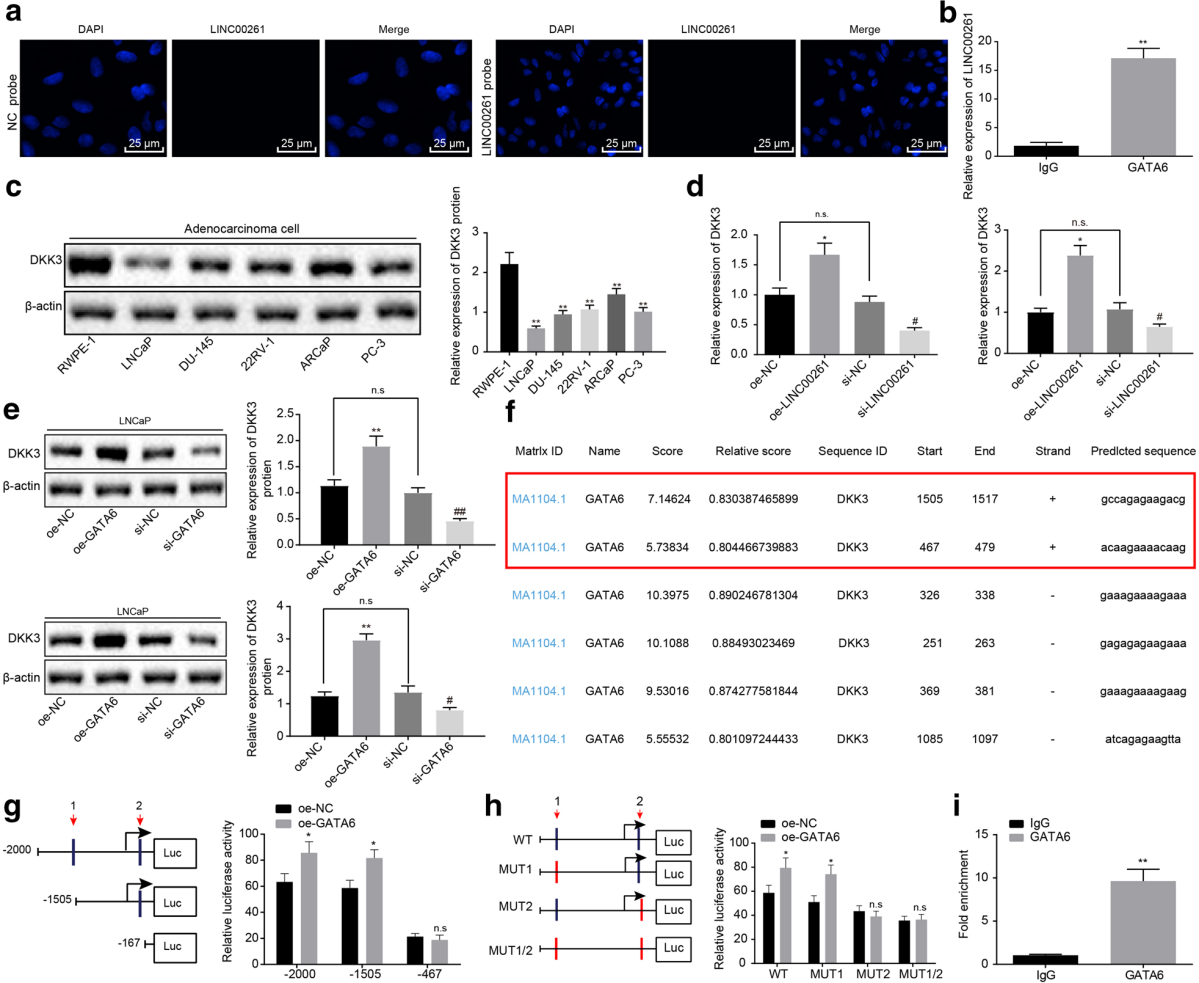
LINC00261 promoted DKK3 expression by recruiting GATA6. **a** The expression and location of LINC00261 measured by RNA-FISH (×400). **b** The binding capacity of GATA6 to the DKK3 protein detected by RIP, * *p* < 0.05 vs. IgG, **p < 0.01 vs. IgG. **c** The expression of DKK3 in all cell lines assessed by Western blot analysis. **d** DKK3 mRNA expression after overexpression or silencing of LINC00261 measured by RT-qPCR, *p < 0.05 vs. oe-NC, ^#^*p* < 0.05 vs. si-NC; **e** The expression of DKK3 protein after overexpression or silencing of LINC00261 measured by Western blot analysis, *p < 0.05 vs. oe-NC, **p < 0.01 vs. oe-NC, ^#^*p* < 0.05 vs. si-NC, ^##^*p* < 0.01 vs. si-NC. **f** Two sites of GATA6 protein and DKK3 DNA identified by website analysis. **g**, **h** The fluorescence intensity in GATA6 and DKK3 promoter detected by dual luciferase reporter gene assay, *p < 0.05 vs. the NC, **p < 0.01 vs. the NC. **i** ChIP assay results; Statistical data are measurement data, and described as mean ± standard deviation, *p < 0.05 vs. IgG, **p < 0.01 vs. IgG. Unpaired *t* test is used to compare data between two groups. The experiment was repeated three times

FISH results showed that LINC00261 was mainly expressed in the nucleus (Fig. 4a). Bioinformatics analysis suggested that LINC00261 may interact with GATA6 and RIP experiment revealed that enrichment of LINC00261 in cells treated with GATA6 was notably increased compared with the cells transfected with IgG (Fig. 4b) (*p* < 0.05), indicating LINC00261 could bind to GATA6.

Western blot analysis was conducted to assess the expression of DKK3 in all prostate cancer and normal cell lines. The expression of DKK3 in prostate cancer cells was the lowest in LNCaP, which was consistent with the trend of LINC00261 expression (Fig. 4c). The results of RT-qPCR and Western blot analysis revealed that compared with cells treated with oe-NC, both mRNA and protein expression of DKK3 in cells transfected with oe-LINC00261 was increased. Furthermore, the mRNA and protein levels of DKK3 in cells after treatment with si-LINC00261 were notably reduced compared with the cells treated with si-NC (*p* < 0.05). Lastly, compared with cells transfected with oe-NC, the DKK3 mRNA and protein levels in cells treated with oe-GATA6 were increased and, compared with cells transfected with si-NC, DKK3 mRNA and protein levels in cells treated with si-GATA6 were decreased (*p* < 0.05) (Fig. 4d, e).

Two potential interaction sites of the GATA6 protein with the DKK3 DNA were obtained by analysis of UCSC (http://genome.ucsc.edu/) and JASPAR (http://jaspar.genereg.net/) (Fig. 4f). The binding sites were verified with a dual-luciferase reporter gene assay and the results showed that compared with cells treated with oe-NC, the ability of GATA6 to activate DKK3 decreased with truncated or mutated form of locus 2 (*p* < 0.05), while the ability of GATA6 activating DKK3 was not affected by truncation or mutation of other sites (*p* > 0.05) (Fig. 4g, h). The results showed that locus 2 was the binding site of GATA6 protein to DKK3 DNA. The binding ability of GATA6 to the DKK3 promoter was then detected by ChIP assay. Compared with IgG, DKK3 DNA bound to GATA6 protein increased (*p* < 0.05) (Fig. 4i, indicating the locus 2 of DKK3 and DNA is the binding site of GATA6 transcription factor. These results suggested that LINC00261 promoted DKK3 transcription expression by recruiting GATA6.

### LINC00261/GATA6 plays a role in prostate cancer cells through DKK3

**Fig. 5:**
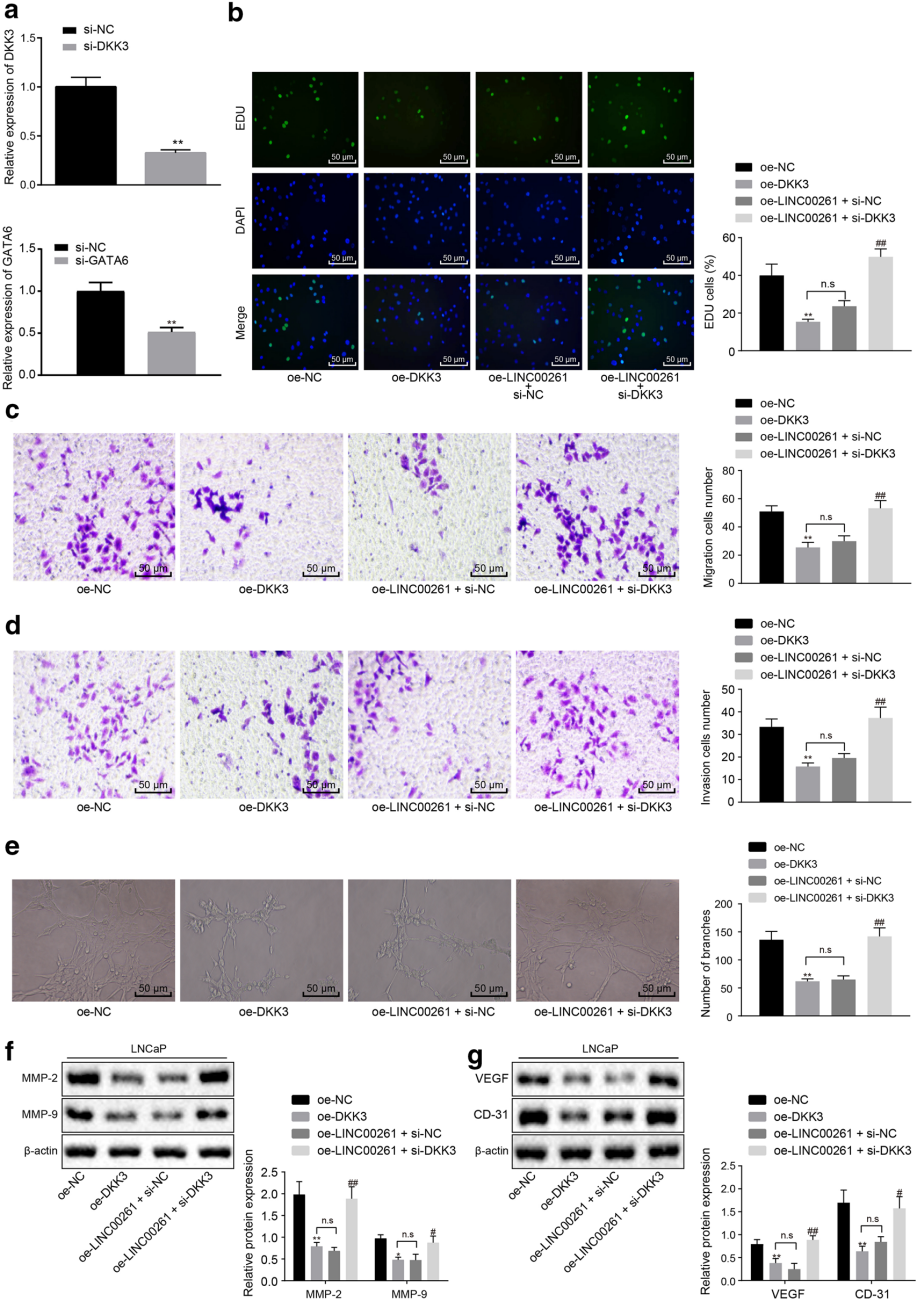
LINC00261 promoted the expression of DKK3 to suppress the metastasis and angiogenesis of prostate cancer by recruiting the transcription factor GATA6. **a** The expression of DKK3 and GATA6 assessed by RT-qPCR. **b** Representative images and statistical analysis of cell proliferation detected by EdU (×200). **c**, **d** Representative images and statistical analysis of cell migration (×200) and invasion (×200) detected by Transwell assay. **e** Representative images and statistical analysis of tube formation (×100). **f**, **g** The protein expression of MMP-2, MMP-9, VEGF and CD31 measured by Western blot analysis. *p < 0.05 vs. oe-NC group, **p < 0.01 vs. oe-NC group, ^#^*p* < 0.05 vs. the cells treated oe-LINC00261 and si-NC, ^##^*p* < 0.01 vs. the cells treated oe-LINC00261 and si-NC. Statistical data are measurement data and described as mean ± standard deviation. Unpaired *t* test is used to compare data between two groups. ANOVA is used to compare data among multiple groups, followed by Tukey’s post hoc test. The experiment was repeated three times

The results of RT-qPCR presented that compared with the cells transfected with si-NC, DKK3 expression in cells transfected with si-DKK3 was decreased, and GATA6 expression in cells transfected with si-GATA6 was also reduced (Fig. 5a). EdU, Transwell and tube formation results (Fig. 5b–e) revealed that compared with the cells after treatment of oe-NC, the proliferation, migration, invasion and tube formation ability of cells transfected with oe-DKK3 was significantly reduced. Also, compared with cells treated with oe-LINC00261 and si-NC, the proliferation, migration, invasion and tube formation ability of cells transfected with the plasmids of oe-LINC00261 and si-DKK3 was significantly increased (*p* < 0.05).

Western blot analysis uncovered that compared with cells transfected with oe-NC, the protein expression of MMP-2 and MMP-9 in cells treated with oe-DKK3 was significantly decreased, and the protein expression of VEGF and CD31 was also decreased (*p* < 0.05). Compared with the cells treated with oe-LINC00261 and si-NC, the protein expression of MMP-2 and MMP-9 in cells transfected with oe-LINC00261 and si-DKK3 was increased, and that of VEGF and CD31 was increased (*p* < 0.05) (Fig. 5f, g). In short, LINC00261 overexpression inhibited the proliferation, migration, invasion and tube formation of prostate cancer cells which could be reversed by DKK3 silencing.

### LINC00261 inhibits the tumorigenicity of prostate cancer cells in vivo

**Fig. 6:**
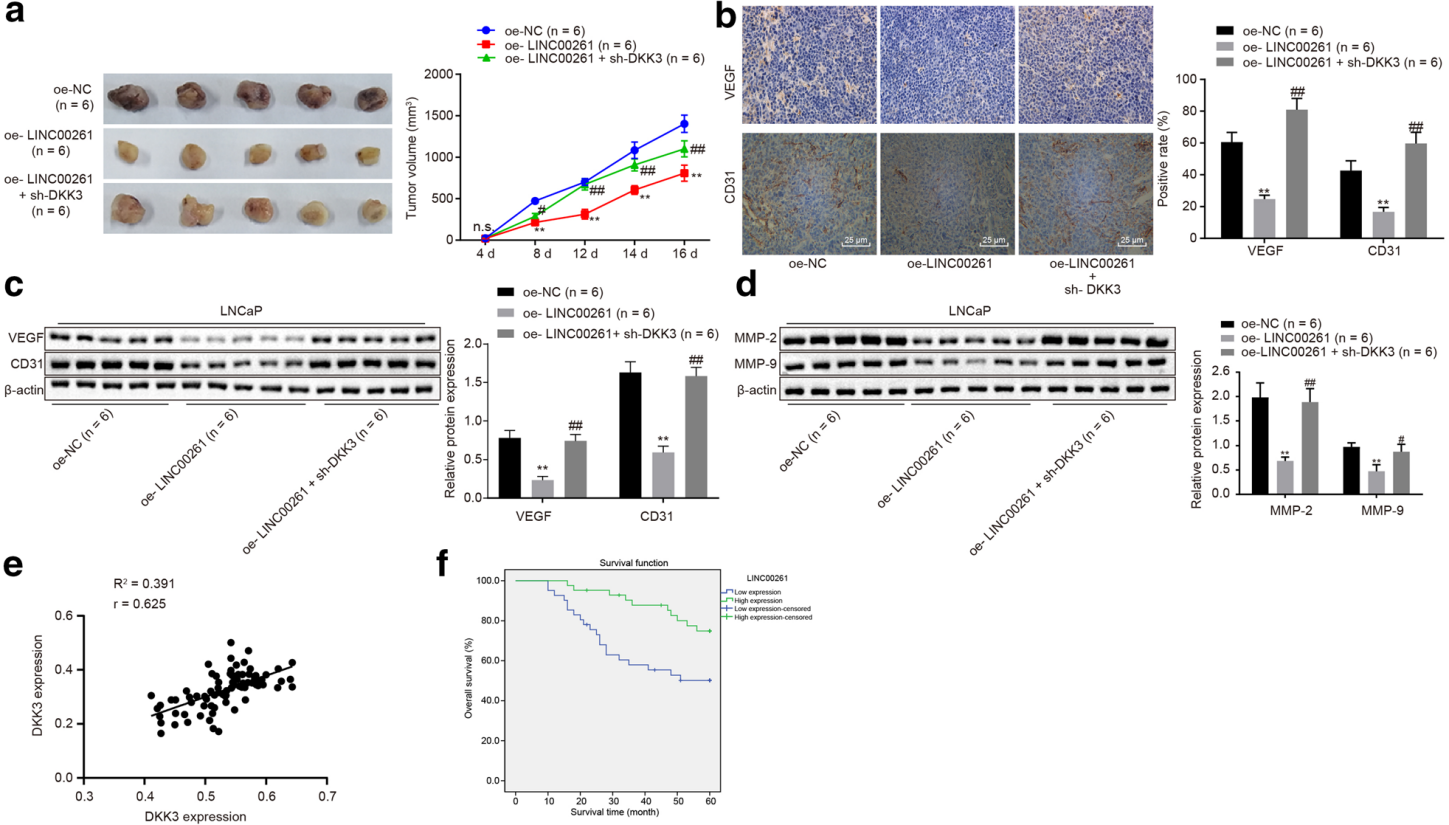
LINC00261 inhibits the growth of prostate cancer cells in vivo. **a** The growth of tumors in *vivo* assessed by tumorigenesis in nude mice. **b** The expression of VEGF and CD31 measured by Immunohistochemistry. **c**, **d** The expression of VEGF, CD31, MMP-2 and MMP-9 assessed by Western blot analysis. *p < 0.05 vs. oe-NC group, **p < 0.01 vs. oe-NC group, ^#^*p* < 0.05 vs. the cells treated oe-LINC00261, ^##^*p* < 0.01 vs. the cells treated oe-LINC00261. **e** The positive correlation between DKK3 and LINC00261 explored by TCGA database. **f** The poor prognosis of patients with low LINC00261 expression. Statistical data are measurement data and described as mean ± standard deviation. ANOVA was used to compare data among multiple groups followed by Bonferroni post hoc test. The experiment repeated three times

The results of tumorigenesis in nude mice supported that there was an obvious difference in tumors from d 12. Compared with the cells treated with oe-NC, the growth of tumors in cells transfected with oe-LINC00261 was slowed down. At the same time, the growth of tumors in cells transfected with oe-LINC00261 and sh-DKK3 showed substantially faster growth compared with the cells treated with oe-LINC00261 (Fig. 6a). These results suggest that overexpression of LINC00261 can inhibit the growth of tumors in vivo.

Immunohistochemical staining showed that the positive expression of VEGF and CD31 was brown. Compared with cells treated with oe-NC, the positive rates of VEGF and CD31 in cells treated with oe-LINC00261 were lower (*p* < 0.05). Compared with the cells treated with oe-LINC00261, the positive rates of VEGF and CD31 in cells transfected with oe-LINC00261 and sh-DKK3 were higher (*p* < 0.05) (Fig. 6b). The expression of VEGF and CD31 was inhibited after LINC00261 overexpression, as measured by Western blot analysis (Fig. 6c). At the same time, the expression of MMP-2 and MMP-9 evaluated by Western blot analysis showed that compared with cells treated with oe-NC, the protein expression of MMP-2 and MMP-9 in cells treated with oe-LINC00261 was decreased (*p* < 0.05), and that was higher in cells transfected with oe-LINC00261 and sh-DKK3 than that in the cells treated with oe-LINC00261 (*p* > 0.05) (Fig. 6d). These findings revealed that overexpression of LINC00261 inhibited the expression of MMP-2, MMP-9, VEGF, and CD34, and suppressed the growth of prostate cancer in vivo. Finally, we collected the prostate cancer data from the database and found that the expression of DKK3 was negatively correlated with the expression of LINC00261, and that patients with high expression of LINC00261 had a poor prognosis (Fig. 6e, f), which was consistent with our experimental results. This suggested that LINC00261 inhibited the tumorigenesis of prostate cancer cells in vivo, and was a potential therapeutic target for prostate cancer.

## Discussion

**Fig. 7:**
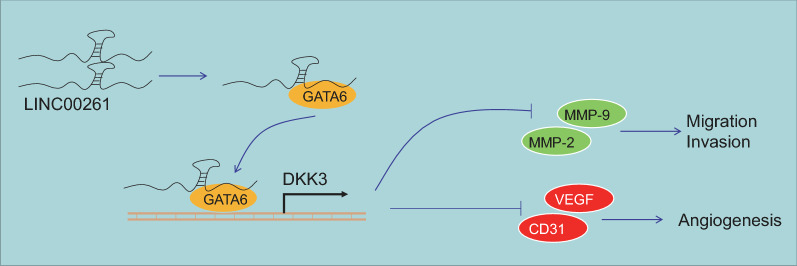
LINC00261 promoted DKK3 expression by recruiting GATA6. LINC00261 reduced expression of MMP-2 and MMP-9, as well as VEGF and CD34, thus suppressing migration, invasion and angiogenesis of prostate cancer cells

Prostate-specific antigen is the most commonly used biomarker for the detection of prostate cancer [20]. However, it is still unfavorable for the early detection of prostate cancer given that the mechanism of prostate cancer development remains unclear. In this study, we find that LINC00261-targeted GATA6 suppresses the proliferation, migration, invasion and angiogenesis of prostate cancer by promoting activation of DKK3 (Fig. 7).

Aberrant expression of lncRNAs is related to tumor progression in many cancers [5]. Previous studies have revealed that lncRNAs are involved in the processes of cell proliferation, migration and invasion [21]. For instance, lncRNA H19 is downregulated in the prostate cancer cell line M12, and the overexpression of H19 suppresses cell migration in prostate cancer cell lines, which suggests H19 may play a suppressive role in prostate cancer progression [22]. Another previous study showed that silencing of lncRNA PCAT29 increases proliferation and migration of prostate cancer cells, and overexpression lncRNA PCAT29 can suppress the growth and metastasis of prostate tumors, indicating that lncRNA PCAT29 is a tumor suppressor in prostate cancer [23]. Also, recent research has revealed that LINC00261 is regarded as a novel biomarker in several cancers. For example, LINC00261 expression is reduced in gastric cancer cells and LINC00261 suppressed cell invasion of gastric cancer, indicating that LINC00261 could be a new biomarker for gastric cancer [24]. In our study, LINC00261 is expressed at a low level in prostate cancer, and LINC00261 negatively correlates with prostate cancer progression. Increasing studies have demonstrated that the aberrant expression of DKK3 is associated with the proliferation and tumor development and it is already considered to be a biomarker and therapeutic target in many cancers [7].

Furthermore, a recent study has already established a relationship between DKK3 and prostate cancer, DKK3 was down-regulated in prostate cancer cells where it inhibited tumor growth, proliferation and migration of prostate cancer [8]. In this study, we proved that DKK3 expression was reduced in prostate cancer cells compared with adjacent normal tissues. In addition, many studies have revealed that aberrant expression of GATA is related to prostate cancer progression [12]. For example, GATA3 expression decreases in prostate cancer cells where it acts as a tumor suppressor [25]. Moreover, compared with non-tumoral liver tissues, GATA6 expression is also reduced in hepatocellular carcinoma tissues, and it was considered to be a potential prognostic biomarker and therapeutic target for liver cancer [26]. These studies demonstrate that GATA6 plays a vital role in the progression of disease. Overexpression of LINC00261 suppressed cell proliferation and invasion in human choriocarcinoma [27] and inhibited migration of trophoblast in pre-eclampsia [28].

Tumor angiogenesis, the process of developing new blood vessels, is known as a critical component of cancer progression, contributing to tumor growth and metastasis [29]. Since VEGF are main molecular drivers of tumour angiogenesis, VEGF signaling pathway inhibitors have been implicated as a therapeutic strategy for prevention of tumors [30]. Herein, we found that overexpression of LINC00261 or DKK3 inhibited cell proliferation and decreased the protein expression of VEGF, and CD31. Furthermore, upregulating LINC00261 expression suppressed the lumen formation of prostate cancer cells as well as inhibiting cell migration and invasion. The RIP results of our study indicated that LINC00261 bound to GATA6. Moreover, Fang et al. showed that silencing of GATA-6 resulted in decreased the expression of important mediators including MMP-2 and MMP-9 [31]. Also, a previous study has shown that GATA4 promoted oncogenesis through suppression of DKK3 expression in hepatoma cells [32]. MMP-2 and MMP-9 are enzymes involved in prostatic development and growth, important to cancer progression [33]. Silencing of DKK-3 has been indicated to activate enzyme activity of MMP-2 and MMP-9, which promotes migration and invasion in prostate epithelial cells [34]. Consist with the previous study, treatment with downregulation of DKK-3 facilitated malignant phenotypes of prostate cancer cells, upregulating MMP-2 and MMP-9, and even reversed the effect caused by overexpression of LINC00261.

## Conclusions

In this study, we found that LINC00261 promoted DKK3 expression by recruiting the transcription factor GATA6. Furthermore, the LINC00261/GATA6/DKK3 axis plays a crucial role in the development and prostate cancer progression; LINC00261-targeted GATA6 suppressed the proliferation, migration, invasion and angiogenesis of prostate cancer by promoting activation of DKK3. LINC00261, GATA6 as well as DKK3 expression in prostate cancer was down-regulated. Overall, our study showed that LINC00261 might offer a novel biomarker for the early detection of prostate cancer. However, a limitation of this study is that only a small number of genes were selected for analysis. We will further explore the effects of DKK3 on prostate cancer by using whole-genome approaches.

## Data Availability

All data generated or analyzed during this study are included in this article.

## Abbreviations

PSA: Prostate-specific antigen
lncRNAs: Long noncoding RNAs
DKK3: Dickkopf-related protein 3
GATA6: GATA binding protein 6
DEGs: Differentially expressed genes
FC: Fold change
TF: Transcription factors
NC: Negative controls
oe-DKK3: DKK3 overexpression
si-DKK3: siRNA targeting DKK3
PVDF: Polyvinylidene fluoride
MMP-2: Matrix metalloproteinase 2
VEGF: Vascular endothelial growth factor
HPR: Horseradish peroxidase
ECL: Electrochemiluminescence
FISH: Fluorescence in situ hybridization
DAPI: 4,6-Diamino-2-phenyl indole
EdU: 5-Ethynyl-2′-deoxyuridine
ChIP: Chromatin immunoprecipitation
RIP: RNA immunoprecipitation
TV: The volume of the tumor
BSA: Bovine serum albumin
DAB: Diaminobenzidine
ANOVA: Analysis of variance
GEPIA: Gene Expression Profiling Interactive Analysis
